# A functional analysis of the CREB signaling pathway using HaloCHIP-chip and high throughput reporter assays

**DOI:** 10.1186/1471-2164-10-497

**Published:** 2009-10-27

**Authors:** Danette D Hartzell, Nathan D Trinklein, Jacqui Mendez, Nancy Murphy, Shelley F Aldred, Keith Wood, Marjeta Urh

**Affiliations:** 1 Promega Corporation, 2800 Woods Hollow Road, Madison, WI 53711, USA; 2 SwitchGear Genomics 1455 Adams Drive, Suite 1317, Menlo Park, CA 94025, USA

## Abstract

**Background:**

Regulation of gene expression is essential for normal development and cellular growth. Transcriptional events are tightly controlled both spatially and temporally by specific DNA-protein interactions. In this study we finely map the genome-wide targets of the CREB protein across all known and predicted human promoters, and characterize the functional consequences of a subset of these binding events using high-throughput reporter assays. To measure CREB binding, we used HaloCHIP, an antibody-free alternative to the ChIP method that utilizes the HaloTag fusion protein, and also high-throughput promoter-luciferase reporter assays, which provide rapid and quantitative screening of promoters for transcriptional activation or repression in living cells.

**Results:**

In analysis of CREB genome-wide binding events using a comprehensive DNA microarray of human promoters, we observe for the first time that CREB has a strong preference for binding at bidirectional promoters and unlike unidirectional promoters, these binding events often occur downstream of transcription start sites. Comparison between HaloCHIP-chip and ChIP-chip data reveal this to be true for both methodologies, indicating it is not a bias of the technology chosen. Transcriptional data obtained from promoter-luciferase reporter arrays also show an unprecedented, high level of activation of CREB-bound promoters in the presence of the co-activator protein TORC1.

**Conclusion:**

These data suggest for the first time that TORC1 provides directional information when CREB is bound at bidirectional promoters and possible pausing of the CREB protein after initial transcriptional activation. Also, this combined approach demonstrates the ability to more broadly characterize CREB protein-DNA interactions wherein not only DNA binding sites are discovered, but also the potential of the promoter sequence to respond to CREB is evaluated.

## Background

Control of gene expression and transcription in mammalian cells is typically achieved through a multi-layered network of protein signaling pathways containing multiple checkpoints to ensure specificity or correct transmission of external stimuli. Regulation of transcriptional activation or repression is crucial for proper development, cell growth, and routine progression through the cell cycle. There is a rapidly growing body of data describing DNA-protein interactions on a genome-wide scale, aided by availability of complete mammalian genome sequences and also the coupling of chromatin immunoprecipitation (ChIP) experiments [1-3] with DNA microarrays analysis (ChIP-chip) [4-10] or ultra high-throughput sequencing (ChIP-Seq) [11-16]. While genome-wide maps of DNA-protein interactions are crucial to understanding global transcriptional networks, understanding the functional consequences of these binding events is equally important. To expand existing approaches to study DNA-protein interactions in living cells, we present two complementary technologies: HaloCHIP, an antibody-free alternative approach to ChIP, for mapping protein binding sites on DNA, and high-throughput reporter assays to measure the promoter activity associated with binding events.

**Figure 1:**
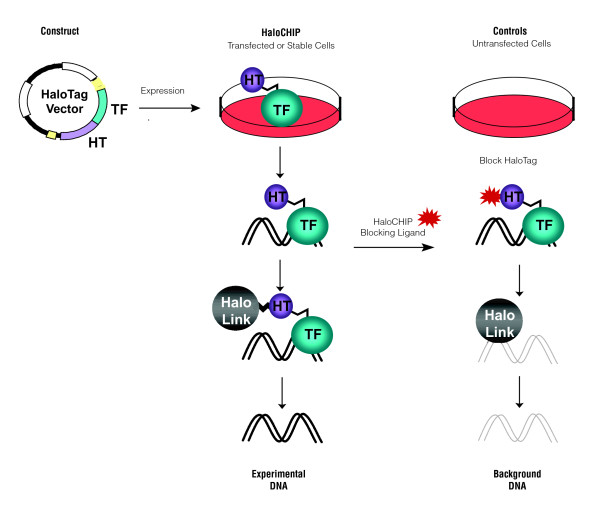
**Schematic of the HaloCHIP process**. The HaloCHIP process is initiated by cloning a desired DNA binding protein-of-interest, i.e. a transcription factor (TF) into a HaloTag (HT) fusion mammalian expression vector. For the experimental HaloCHIP sample, the HaloTag fusion protein is expressed in the desired cell line and then crosslinked to DNA *in vivo *with formaldehyde. Treated cells are lysed, sonicated to shear the chromatin, and incubated with HaloLink resin, which directly and covalently captures all crosslinked HaloTag-fusion complexes. The resin is stringently washed to remove non-specific proteins and DNA, and captured regions of DNA are released by reversal of the crosslinks. The resultant DNA can be further purified for downstream analysis. Two possible controls, which provide an estimate of background DNA capture, are recommended for the HaloCHIP process. The first, referred to in the text as the untransfected control, involves the use of untransfected cells which are processed in parallel with the HaloCHIP experimental sample. The second, referred to as the blocking ligand control, is generated by splitting the cell lysate equally into separate tubes prior to incubation with the HaloLink resin and incubating the control sample only with a fluorescent HaloCHIP blocking ligand, preventing interaction of the HaloTag complexes with the HaloLink resin during the subsequent incubation step.

The success of ChIP relies heavily on the success of the immunoprecipitation step in the process, creating a need for alternative approaches when the antibody against the DNA binding protein is either not functional or available for the ChIP assay [17-21]. Such alternative approaches are derived from the standard ChIP method and include the initial formaldehyde crosslinking of protein:DNA complexes, yet typically differ in the use of protein fusion tags, which allow for complexes to be isolated using either an antibody against the tag [17,21] or direct capture on a resin that interacts with the fusion tag [18-20]. The latter is the basis for the HaloCHIP method, which utilizes the HaloTag protein [19,20], a 33 kDa protein fusion tag, that can be cloned N- or C-terminally to a DNA binding protein of interest[19,20] (Figure 1). In the HaloCHIP method, the HaloTag fusion protein is expressed either transiently or stably in mammalian cells and crosslinked complexes can be directly captured from a cellular lysate via covalent binding to a HaloTag-specific resin, termed HaloLink [19,20] (Figure 1). The complete covalent linkage established at this point allows for extensive washing to remove non-specific protein and DNA, followed by standard reversal of the crosslinks to release the DNA fragments which were bound to the DNA binding protein (Figure 1). Several controls for the HaloCHIP method are possible to show that capture is specific in this process and provide an excellent estimate of background (Figure 1).

Both the HaloCHIP and ChIP method yield information about the location and timing of binding events on DNA, but do not provide information as to the cellular response or consequence of the given binding event. Currently, mRNA and protein levels are measured to determine whether or not a gene has been activated or repressed, but a more direct measure of the transcription potential or function of bound DNA sequences would be ideal. To complement these approaches and also increase sensitivity, high-throughput reporter assays can be used (Cooper et al. 2006). High-throughput reporter assays utilize a 384-well format that enable the functional measure of thousands of endogenous human promoters. Each individual promoter is fused to a luciferase reporter gene and transiently delivered to living cells. Upon protein binding to the promoter region, the luciferase reporter gene is activated and the degree of this activation can be quantitatively determined before and after a stimulus by measuring the light output. This allows real-time monitoring of transcriptional activation or repression from the promoter-reporter construct after stimulus of a pathway or response to other cellular conditions.

To demonstrate the use of these approaches to further understanding of DNA-protein interactions in living cells, we chose to study the CREB transcription factor [22-25]. The model system of the CREB signaling pathway has been elegantly studied and its binding targets have been described previously at the level of individual promoters as well as a genome-wide scale [12,22,26-29]. CREB belongs to a family of transcription factors including activating transcription factor 1 (ATF1) and the cAMP response element modulator (CREM), which regulate gene expression in response to changes in cAMP and other cellular signals [23,24]. Upon activation of the protein kinase A pathway or stimulation of other kinases, CREB is directly phosphorylated on several critical serines [22,30], though phosphorylation is not required for binding to DNA [31]. The phosphorylation events instead allow subsequent recruitment and binding of transcriptional co-activators CREB binding protein (CBP)/p300 as well as transducers of regulated CREB (TORCs) to the promoter region [32-35]. Previous studies have shown that CREB co-factors are often necessary for transcription activation and that CREB binding to DNA, even in its phosphorylated form, is not usually sufficient to activate transcription [34-39].

In this paper, CREB binding is mapped at a much higher resolution than previous studies and covers all known and predicted human promoters using the HaloCHIP method in conjunction with DNA microarrays, ("HaloCHIP-chip"). As this is a new approach for studying genome-wide protein:DNA interactions, these data were compared to the standard CREB ChIP-chip process using an antibody against the endogenous CREB protein, revealing a high degree of overlap between the methods and also to previously published data [26,29]. To further correlate DNA binding events to potential transcription activation or repression, a subset of CREB-bound promoters were analyzed using high-throughput reporter assays in the presence or absence of protein kinase A pathway activators as well as the CREB transcriptional co-activator, TORC1. All together these data reveal new CREB-bound promoters and binding preferences on DNA, interesting functional activities provided by the high-throughput reporter assays, and new insights into CREB-mediated transcription regulation.

## Results

### Specific binding and enrichment of CREB promoters in HaloCHIP

**Figure 2:**
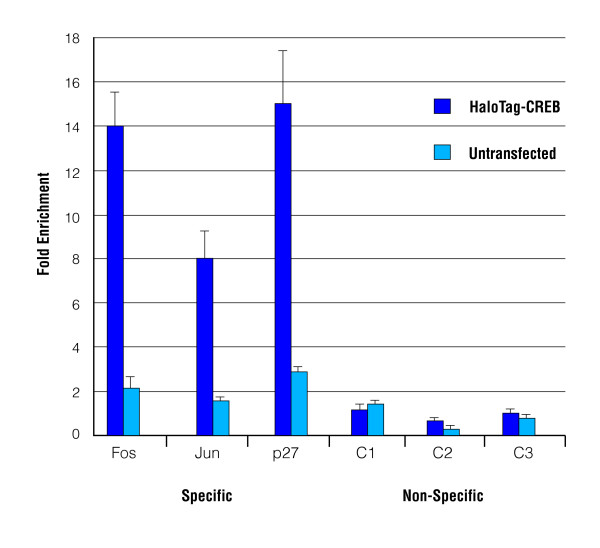
**Specific DNA binding of HaloTag-CREB *in vivo***. HaloCHIP experiments were performed in triplicates, on HeLa cells transiently expressing HaloTag-CREB or untransfected as a control. Resulting DNA from both the HaloTag-CREB and Untransfected control sample was amplified and analyzed using Plexor quantitative PCR. Total amounts of DNA for both samples were calculated for three promoters which CREB is known to bind [28], Fos, Jun, and p27, as well as three control sequences, C1, C2, and C3, which do not contain CRE consensus binding sites. Depicted in dark blue is the fold enrichment of each CREB-specific promoter over the average amount of the three control promoters for the HaloTag-CREB HaloCHIP experimental sample. In light blue is the identical calculation for the Untransfected HaloCHIP control sample.

The significant advantages of the HaloCHIP method are the direct and covalent capture of the crosslinked DNA-protein complexes on the HaloLink resin, eliminating the need of an antibody and preventing loss or diffusion of complexes after capture (Figure 1). As with the use of any fusion tag, it is important to show the tagged protein behaves similar to the endogenous protein. Previous studies with HaloTag fusion proteins have demonstrated proper physiology, including DNA binding and localization [19,40-45]. In order to demonstrate specific DNA binding of the HaloTag-CREB fusion protein, HaloCHIP assays were performed in triplicates using transiently expressed HaloTag-CREB as the experimental sample and untransfected HeLa cells as a control. The relative abundance of three known CREB-specific promoters, Fos, Jun, and p27, [24,28] and three negative control sequences, called C1, C2, and C3, which are non-genic regions from the human genome that lack CRE sites, were then analyzed using Plexor quantitative PCR (Figure 2). The CREB-specific promoters show an average enrichment of 12.5 fold, while the untransfected control showed an enrichment of 1.8 fold (Figure 2). The negative control sequences are not enriched in either sample (Figure 2) Similar results were obtained using the HaloCHIP blocking ligand (Figure 1) as a control (data not shown). After this initial validation of specific binding, the HaloCHIP DNA was then prepared for hybridization to a human promoter microarray to ascertain binding on a genome-wide scale.

### HaloCHIP-chip DNA oligo array design

**Figure 3:**
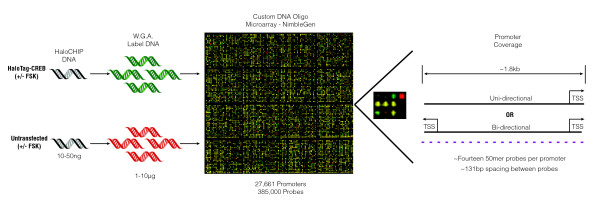
**Schematic of the HaloCHIP-chip microarray experiment design**. HaloCHIP DNA (10-50 ng) obtained for both the experimental HaloTag-CREB and untransfected control sample was purified and amplified to a concentration of 1-10 μg using the whole genome amplification (WGA) method (Sigma) [46]. The HaloTag-CREB amplified sample was labelled with Cy5 (green) and the untransfected control sample with Cy3 (red), then hybridized to a custom DNA oligo microarray manufactured by Roche NimbleGen. The oligo array was designed to cover on average a 1.8 kb region of 27,661 human promoter regions that contain 33,255 TSS predicted by SwitchGear Genomics. To obtain coverage of each promoter, an average of fourteen 50mer single stranded DNA probes, shown in purple, per promoter were used, with an average spacing of 131 bp per probe.

To measure the genome-wide binding sites of CREB and to assess specificity of binding on a global scale, CREB HaloCHIP DNA was hybridized to a custom DNA oligo microarray. In the HaloCHIP-chip strategy, two samples, the experimental and untransfected control, were each treated with or without forskolin (FSK) and processed through HaloCHIP (Figure 3). Similar to ChIP, the HaloCHIP derived DNA from each sample, typically 10-100 ng/reaction, required subsequent amplification to obtain amounts sufficient (1-10 μg) for microarray analysis. The whole genome amplification (WGA) method was used for amplification [46], and the experimental and control samples were labelled with Cy5 and Cy3, respectively (Figure 3). A custom DNA oligo microarray was designed based upon promoter regions defined by SwitchGear Genomics genome-wide set of predicted transcription start sites (Figure 3) [47-50]. An average of 14 probes, each 50-mers, was designed to span a 1.8 kb region of each known promoter region. The probes were chosen to sequences primarily upstream of a transcription start site, with an average of 131 bp spacing between probes. In total, approximately 385,000 probes were used to cover 27,661 promoter regions, including 33,255 transcription start sites (TSS) (Figure 3).

### CREB HaloCHIP-chip array data analysis and cut-offs

A total of 7 independent CREB HaloCHIP-chip experiments were conducted; 4 were treated with forskolin and 3 without. All showed significant enrichment, however very few differences were detected between the untreated and FSK treated samples, as was seen in previously published data [29]. The log2 ratios obtained for each promoter were averaged across the 7 independent array experiments to obtain a single value for each promoter region. To calculate the positive and negative predictive values (PPV and NPV respectively) of the array data at different percentile thresholds, the promoters were sorted based on their average log2 ratio for enrichment and validated by qPCR. For the PPV determination, 12 sequences each were chosen from the Top 1%, 5%, and 10% of the sorted list and for the NPV determination, 36 sequences were randomly chosen from the bottom 50%. The Top 1%, 5%, and 10% categories showed PPVs of 100%, 92%, and 50%, respectively (Table 1), while the NPV from the Bottom 50% was calculated to be 94.5% (Table 1). These values indicate the high quality of the data, and based upon the PPV calculations, the cut-off for calling a promoter "CREB-bound" in subsequent analyses was chosen to be the Top 5% of the list, corresponding to 1,383 promoters.

**Table 1:** CREB HaloCHIP-chip positive and negative predictive values.

**Positive category**	**Enriched promoters**	**PPV**
Top 1%	12 of 12	100%
Top 5%	11 of 12	92%
Top 10%	6 of 12	50%
		
**Negative category**	**Enriched promoters**	**NPV**
Bottom 50%	2 of 26	94.50%

Twelve promoters each from the Top 1%, Top 5%, and Top 10% categories of the HaloCHIP-chip data were chosen to determine the positive predictive value and 36 promoters were chosen from the Bottom 50% of the list for determination of the negative predictive value. SYBR green qPCR was used to determine the presence of these promoters in triplicates samples of HT-CREB experimental and untransfected control HaloCHIP DNA and promoters were considered enriched if having a signal:background ratio ≥ 2-fold between the experimental and control sample. Based upon these results, further analysis of the HaloCHIP-chip data was limited to promoters identified within the Top 5% of the list.

### Comparison of CREB HaloCHIP-chip and ChIP-chip array data

To compare the binding of HaloTag-CREB to the physiological expected binding pattern of the endogenous CREB protein, a conventional ChIP-chip experiment using an antibody to CREB in HeLa cells and using the same oligo DNA microarray was performed. The Top 1% (277) and Top 5% (1383) of promoters showed significant overlap, 35% and 45% respectively, between HaloCHIP and ChIP-chip, representing a 44-fold and 10-fold over-representation of overlap compared to what would be predicted by chance (Table 2). This degree of overlap is within the range of similarity to what is seen between biological replicates of either HaloCHIP-chip or ChIP-chip data sets, indicating that the two methods are yielding equivalent results within the limitations of experimental error of the overall process (Table 2) [11,51].

**Table 2:** Comparison of CREB HaloCHIP-chip and CREB ChIP-chip data.

**HaloCHIP-chip:ChIP-chip compared**	**Number of Promoters**	**Percentage Overlap**	**Enrichment over Random**
Top 1%	96/277	35%	44
Top 5%	616/1383	45%	10
Top 1%*	47/182	26%	5
Top 5%*	212/898	24%	5

**HaloCHIP-chip:HaloCHIP-chip compared**	**Number of Promoters**	**Percentage Overlap**	**Enrichment over Random**

Top 1%	140/277	51%	65
Top 5%	734/1383	53%	11

**ChIP-chip:ChIP-chip compared**	**Number of Promoters**	**Percentage Overlap**	**Enrichment over Random**

Top 1%	115/277	42%	53
Top 5%	477/1383	34%	7

The number of promoters found to overlap between the CREB HaloCHIP-chip and standard ChIP-chip experiments were determined for both the Top 1% and 5% of each respective list. Also shown is the calculation of the fold enrichment over what is expected for a randomized comparison. The first Top 1 and 5% overlap is between CREB HaloCHIP-chip and ChIP-chip data obtained from the same probes and arrays, therefore the number of promoters, 277 and 1383 respectively, used for comparison is the same between the two lists. The (*) indicates the comparison of the HaloCHIP-chip array data to previously published Top 1 and 5% CREB ChIP-chip data [29]. The Top 1 and 5% CREB ChIP-chip data correspond to 182 and 898 promoters respectively, and it was these which were used for the comparison to the Top 1 and 5% of the HaloCHIP-chip array data. Calculated in a similar fashion is the percentage overlap and enrichment over random between HaloCHIP-chip biological replicate sets or ChIP-chip biological replicate data sets.

Comparisons were also performed to between HaloCHIP-chip and previously published CREB ChIP-chip data (Table 2) [29]. As the CREB ChIP-chip DNA microarray covered approximately 8,000 fewer number of promoters, the Top 1% and Top 5% of the list correspond to 182 and 898 promoters [29], respectively, which were then used for the comparison (Table 2). A slightly lower overlap of 26% and 23.8%, respectively is observed, corresponding to a 5-fold over-representation for both categories compared to what would be predicted by chance (Table 2). This is not surprising given the differences between these experiments including; the cell lines used, method of amplification for the array, as well as the different array design and platform [29]. Nevertheless, given these differences it was very encouraging to see a significant overlap between these independent results.

Further support that the promoters identified by the CREB HaloCHIP-chip approach are specific for CREB function, comes from Gene Ontology (GO) analysis of the Top 1% promoters. GO analysis shows clusters of promoters found which are involved with histone assembly, chromatin architecture, RNA and DNA metabolism, and nucleic acid binding pathways, all cellular processes which CREB has been shown to regulate (Table 3) [23,24].

**Table 3:** Gene Ontology analysis of CREB HaloCHIP-chip promoters.

**Cellular Functions**	**Number of Promoters**	**p-value**
Histone Assembly	12 of 65	1.26E-06
Chromatin architecture	20 of 261	7.63E-07
Ribonucleic Complexes	26 of 392	7.06E-07
RNA processing	26 of 395	8.01E-07
DNA metabolism	38 of 638	2.93E-08
Nucleic acid binding	110 of 2764	2.19E-09

The Top 1% of the CREB HaloCHIP-chip promoters (277) was analyzed using Gene Ontology (GO) and the different categories in terms of cellular function of promoters within the Top1%. Also shown is the number of CREB HaloCHIP-chip promoters found as compared to the number of known human promoters within each category and the corresponding p-value calculated by GO.

### High resolution mapping of CREB binding sites relative to endogenous transcription start sites

Previous genome-wide CREB ChIP-chip studies were conducted on spotted PCR product microarrays [26,29]. Our use of a custom oligo array that tiles across extended promoter regions (Figure 3) gives us the unique ability to map CREB binding events at much higher resolution that in turn allows us to determine the precise location of binding events relative to transcription start sites. First, we examined the occurrence and location of putative CREs in the HaloCHIP dataset as a whole. Enrichment of binding to both full and half CRE consensus sites within the Top 1% and 5% is observed as compared to the Bottom 90% of the array list (Table 4). Also, full and half CRE sites are highly over-represented in the region 100 bp upstream of the transcription start site (data not shown), consistent with results seen previously studying the binding pattern of endogenous CREB [26,29].

**Table 4:** Match of full and half CRE consensus sites in CREB HaloCHIP-chip data.

**Categories**	**% with Full CRE site**	**% with Half CRE Site**
Top 1%	22%	89%
Top 5%	12%	86%
Bottom 90%	3%	57%

CREB HaloCHIP-chip promoters from the Top 1%, Top 5%, and Bottom 90% categories were analyzed for the presence of the full CRE or half CRE consensus site and number of promoters containing these sequences are depicted. A full CRE site was defined as a perfect match to the consensus TGACGTCA. A half CRE site was defined as a perfect match to the consensus TGACG or CGTCA.

**Figure 4:**
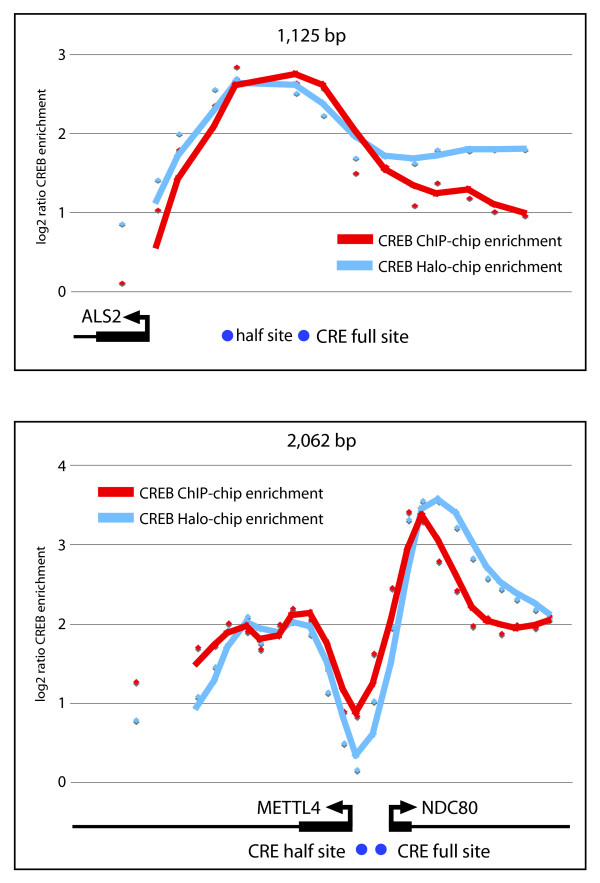
**High-resolution analysis of CREB binding at uni-directional and bi-directional promoters**. Depicted are CREB binding data from two representative promoter regions, the length (bp) of each is indicated above the graph. The log2 ratio for each probe spanning the promoter region was determined for the CREB ChIP-chip (plotted in red) or HaloCHIP-chip (plotted in blue) data. Positions of transcription start sites (TSS) are shown with arrows indicating the direction of transcription with length of exons in blocks and introns drawn as lines. Positions of full and half CRE sites relative to the location within the promoter region are indicated below by blue dots. **A**. Example of CREB binding to a unidirectional promoter, ALS2, where peak binding is observed upstream of the TSS and localized to the CRE consensus sites. **B**. Example of CREB binding to a bidirectional promoter, METTL4 and NDC80, where peak enrichment is located downstream of the TSSs, but the peak enrichment does not localize to CRE consensus sites.

The design of the array also allowed for further in-depth analysis of binding to both unidirectional and bidirectional promoters. A striking observation was that the majority of CREB-binding events were at bidirectional promoters, with 60.7% and 53.7% of total promoters being bidirectional in the Top 1% and 5%, respectively. Previous work has shown that approximately 10% of the genes in the genome are divergently transcribed and regulated by a bidirectional promoter (transcription start sites separated by less than 1000 bp) [49,52,53], suggesting a strong preference of CREB for binding to divergently transcribed genes. Of the 27,661 promoters covered in this study, we estimate that 19% have evidence for oppositely transcribed transcripts initiating in that region. An example of CREB binding at a unidirectional promoter, defined as a promoter associated with a single transcription start site, is shown in Figure 4a. As expected for both the HaloTag and endogenous CREB, there is enrichment of binding directly upstream of the transcription start site around the location of a putative CRE site (Figure 4a), and for our subset analyzed, this pattern of binding was observed greater than 60% of the time. Analysis of binding within bidirectional promoters revealed interesting binding patterns for CREB. As shown in Figure 4b, two distinct peaks of high enrichment are observed just downstream of the transcription start sites of both genes within a bidirectional promoter, while no enrichment is observed in the intergenic space between the transcription start sites. As this is observed for both the HaloTag-CREB fusion protein as well as the endogenous CREB protein, it does not appear to be an artefact of either the HaloCHIP or ChIP methodology. It was also surprising to see that highest enrichment was not localized at the location of putative CRE sites in bidirectional promoters, suggesting CREB is either bound directly elsewhere on DNA or crosslinking to complexes not bound to CRE consensus sites (Figure 4b).

### CREB high-throughput reporter assay analysis

**Figure 5:**
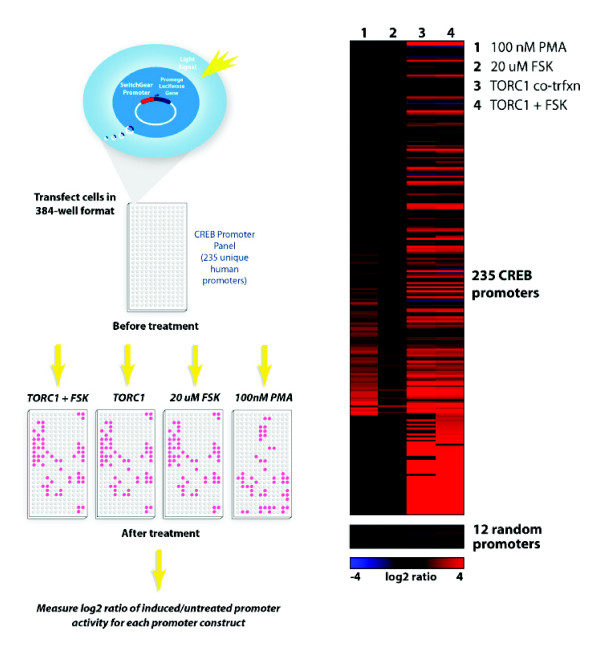
**High-throughput reporter assays of CREB-bound promoters**. **A**. Experimental design of high-throughput reporter assays. A schematic showing the high-throughput reporter assay experimental design. Promoters are fused to luciferase, transfected in a desired cell line, and stimulated under different conditions. Luciferase activity is measure from uninduced and induced samples and the log2 ratio of these differences is calculated. **B**. Heatmap of inducible promoter activity. A total of 235 promoters, chosen from CREB ChIP-chip and HaloCHIP-chip data, along with twelve negative control promoters were fused to the luciferase gene, transfected into HeLa cells, and treated with stimulants; forskolin (FSK), PMA, or co-transfected with a transcriptional co-activator, TORC1 +/- FSK. Each box represents the log2 ratio of induced/untreated for each promoter in each condition where the intensity of red is proportional to the strongest induction and the intensity of blue is proportional to the strongest repression. The presence of TORC1 by co-transfection shows the highest number of promoters induced to the highest degree. The box of 12 control promoters at the bottom of the panel are random promoters from the genome and show very little inducible activity in any of the conditions tested.

The combination of the CREB genome-wide binding events identified in this study along with those reported previously confirm CREB binding events, however do not predict the transcriptional regulation of its target genes. Furthermore, CREB binding was measured in a limited set of conditions (with and without forskolin stimulation), and it is known that the CREB pathway is activated by a wide variety of cellular and environmental stimuli [23,24]. To further characterize the CREB binding events, we assembled a collection of cloned human promoters (1 kb on average) in a luciferase reporter vector that represented a subset (235) of known CREB targets previously identified [26,27,29]. As predicted from the earlier overlap calculations with the CREB ChIP-chip data (Table 2) approximately 35% of these promoters (84) were identified in the Top 5% of the HaloCHIP-chip array data. As a control, 12 random promoters that were not targets of CREB were also fused to luciferase reporters (Figure 5a). Utilizing our high-throughput reporter assay platform, we measured the activity of each of these fragments in HeLa cells in triplicate in 5 different conditions: no treatment, forskolin (FSK) stimulation, phorbol 12-myristate-13-acetate (PMA) stimulation, and co-transfection with a TORC1 expression construct with and without FSK stimulation (Figure 5a). We considered a promoter induced if the absolute activity was significantly above background, passed a T-test at p < 0.05, and had a magnitude of change greater than 2-fold.

The promoter macroarray results are summarized in Figure 5b, and the percentage of the total CREB promoters tested (235), as well as the HaloCHIP subset (84), that were induced in the different conditions were determined (Table 5). For both the CREB set and the HaloCHIP subset, only ~10% were induced by FSK treatment alone (Figure 5b, Table 5). This is not surprising since it is known that many other factors are necessary for the transcriptional activation of CREB-bound promoters and both our binding data and previously reported data did not show an appreciable difference in DNA binding between +/- FSK treatment [23,29,35]. Indeed, the TORC1 co-transfections induced more than 50% of the constructs (Table 5) and also conferred the largest fold-changes in activity (Figure 5b) highlighting the importance of co-factors in the transcriptional activation of CREB-bound promoters [32,34]. Similar trends were observed between the overall CREB set of promoters as compared to the HaloCHIP subset, indicating those identified by HaloCHIP respond similarly to CREB-specific stimuli (Table 5). The 12 random promoters from the human genome do not show induced activity in any of the conditions tested, indicating that the false positive rate of the reporter assay for CREB activity is very low (Figure 5b).

**Table 5:** Percentage of promoters activated in various conditions in functional macroarrays.

	**Total CREB Set Tested (235)**	**HaloCHIP Subset (84)**
**Stimulants**	**% Induced**	**% Induced**
FSK	9.8%	9.5%
PMA	31.9%	26.2%
TORC1	60.4%	50.0%
TORC1 + FSK	65.1%	54.8%
ANY	76.2%	66.7%

Table 5 shows the percentage of promoters activated in each condition from both the total CREB set (235) or a subset of the total CREB set (84), termed the HaloCHIP subset, that was found in the Top 5% of the CREB HaloCHIP-chip array list. The HaloCHIP-chip data follows the same trend of response to each stimulus as the larger CREB set.

Given the interesting CREB binding patterns at bidirectional promoters (Figure 4b), we looked specifically at the promoter activities of bidirectional promoters in our reporter assay dataset. There were a total of 7 bidirectional gene pairs for which we collected promoter activity data for each direction. The majority of the pairs showed very low activity in both directions in the untreated cells suggesting that CREB-bound bidirectional promoters are not transcriptionally active in an un-induced state. Two of the 7 bidirectional gene pairs, which regulate two pairs of histone genes, had constitutively high promoter activities in both directions, irrespective of stimulation conditions.

**Figure 6:**
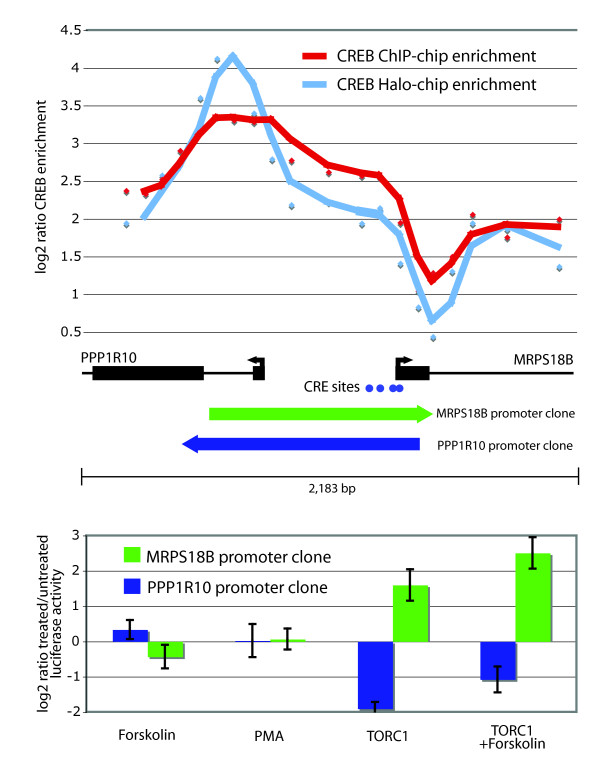
**CREB binding and promoter activity at a bidirectional promoter showing opposite induction patterns in the presence of TORC1**. **A**. CREB binding to PPP1R10/MRPS18B bidirectional promoter. Identical to Figure 4, the log2 ratio for each probe spanning this bidirectional promoter was determined for the CREB ChIP-chip (plotted in red) or HaloCHIP-chip (plotted in blue) data. Spacing between probes is approximately 131 bp. Positions of transcription start sites (TSS) are shown with arrows indicating the direction of transcription with lengths of exons in blocks and introns drawn as lines. Positions of full CRE sites are indicated below by blue dots. Also shown is the region of each cloned promoter fragments, indicated by the green and blue arrows, used in the luciferase assay shown below in panel B. The arrows indicate the direction in which the cloned fragments were tested in the luciferase assay. **B**. The log2 ratio of treated/untreated luciferase activity is plotted for each promoter fragment in each condition indicated. The MRPS18B promoter (in green) shows significant inducible activity in the presence of TORC1 with and without FSK, while the PPP1R10 promoter (in blue) shows significant repression in the presence of TORC1 with and without FSK.

The most interesting example was seen for two of the bidirectional promoter pairs, depicted in Figure 6a, b. For these bidirectional promoters, no induction was observed with either FSK or PMA, but in the presence of TORC1, promoter activity in one direction was significantly up-regulated while promoter activity was repressed in the other direction (Figure 6b). CREB enrichment at the MRPS18B promoter was observed at CRE sites just upstream of the TSS, while enrichment to the PP1R10 promoter was downstream the TSS and not associated with a CRE site (Figure 6a). Interestingly, only the MRPS18B promoter shows transcriptional activation in the reporter assay, while the PPP1R10 promoter is significantly repressed in the presence of TORC1 and FSK (Figure 6b). These results suggest that TORC1 plays an important role at some promoters in determining the direction of transcriptional activation.

## Discussion

We applied here two technologies, HaloCHIP and high-throughput promoter assays, to study and more fully characterize the CREB transcription pathway than previously done. The HaloCHIP method (Figure 1) offers an alternative approach for the capture of intracellular DNA-protein complexes and was developed to address the challenges of antibodies required for the existing ChIP method. The use of a robust protein tag eliminates the need for a qualified antibody and enables researchers to study highly similar paralogs, different isoforms, or point mutants of a transcription factor that may not be distinguishable by an antibody. Also, due to rapid and covalent binding kinetics HaloTag with its ligands, protein complexes can be captured efficiently from dilute solutions without concern of loss due to diffusion off the resin, allowing for the use of a much smaller number of cells (2-4 × 10^5^) per HaloCHIP experiment as compared to the standard ChIP experiment (~1 × 10^7^) [4,10]. As with the use of any protein fusion tag for ChIP or HaloCHIP experiments there are concerns as to potential alteration of DNA binding due to interference by the fusion tag or changes in expression level. The CREB HaloCHIP-chip results show that binding to DNA on a genomic-scale was specific for the CREB protein and had a significant degree of overlap with conventional CREB ChIP-chip data, suggesting the HaloTag-CREB fusion protein is binding to DNA similarly to the endogenous CREB protein.

In addition to identification of new CREB-bound promoters with these array studies, we extended our studies of the CREB pathway by measuring the functional activity of over 200 CREB-target promoters [26,27,29] in a high-throughput reporter assay experiment (Figure 6a). Many diverse responses are regulated through the CREB pathway and unique subsets of CREB-bound genes may be transcriptionally activated and responsive to particular stimuli. The high-throughput reporter assays of CREB-bound promoters gives the ability to stratify CREB binding events based on the transcriptional activity of the fragments of DNA to which they bind. Analysis of the CREB pathway using the functional promoter macroarrays revealed only a small percentage of promoters were responsive to FSK, correlating well with the HaloCHIP-chip data showing minimal changes in binding between untreated and FSK treated cells. Interestingly, a much larger percentage of promoters were responsive to PMA, and an even greater percentage to the TORC proteins. These functional results provide further support for the idea that co-factors are a crucial part of the CREB signaling pathway and while reporter assays lack the full chromatin context of the genome, by using extended promoters regions that are 1 kb in length, we were able to observe transcriptional effects by co-factors, which may interact with proximal sites. In order to analyze CREB-enriched sites at a much higher resolution than was previously performed, custom oligo microarrays were used. This detailed analysis provided interesting and novel insight into the localization of CREB at the promoters of genes. A simple assumption is that the experimental enrichment for CREB binding would be coincident with the location of the CRE. Indeed this was observed for the majority of unidirectional promoters (Figure 4a). However, a distinctly different pattern is observed for a large fraction of bidirectional promoters, where the peaks of highest enrichment are seen downstream of the closest TSS, often not coinciding with the location of CREs (Figure 4b). This pattern was seen consistently for many bidirectional promoters, in both HaloCHIP-chip and ChIP-chip data sets, indicating this is not a phenomenon associated with a particular method.

These particular binding results suggest a number of interesting scenarios for which additional experiments will be needed. It may be the case that there is a secondary structure of the CREB-DNA complex at bidirectional promoters where the peaks of enrichment reflect the higher order crosslinked structure rather than the true localization of the CREB protein on the linear strand of genomic DNA. An alternative explanation is that CREB may be a part of a paused transcription initiation complex. In this scenario, CREB could initially bind upstream of the TSS in the bidirectional promoters, form its known interactions with the RNA PolII complex, move after initiation with the complex, and then pause at particular sites downstream of the TSS. Recent work has shown that a paused transcriptional complex containing transcriptional regulators are more abundant than previously thought [54-56], and this explanation would produce the enrichment pattern that we observe for CREB at bidirectional promoters (Figure 4b, 6b).

Results from the promoter reporter assays for bidirectional promoters are also consistent with this scenario, since a paused transcriptional machinery would likely result in lower reporter activity as was seen for the majority of bidirectional promoters tested. Perhaps most interesting is the functional behaviour of a subset of the bidirectional promoters in the presence of TORC1. In 2 out of 7 cases tested, the activity of a bidirectional promoter was strongly induced in one direction and strongly repressed in the opposite direction in the presence of TORC1. The strongly repressed promoters show CREB binding which is downstream the TSS, while the strongly induced promoters show expected upstream promoter binding. Also, the ability of the TORC1 protein to differentially regulate promoter activity, suggests that CREB co-factors may also help to regulate the directionality of transcription. This is particularly relevant for the CREB transcription factor, since over 50% of CREB binding sites are located in bidirectional promoters as we have reported for the first time.

## Conclusion

This broad survey of the transcriptional activity of CREB-bound promoters provides a valuable layer of functional data for the CREB protein. Future efforts to compare the activity of these promoters in many more conditions will help to further understand CREB signaling and mutational analysis of the bidirectional class of CREB-bound promoters will help to dissect the mechanism of bidirectional gene regulation. The use of the new technologies presented here however is not limited to the study of the CREB pathway, rather can be generalized to study any transcriptional pathway. The HaloCHIP method, like the standard ChIP process, can be used to study DNA binding both on a small scale, as well as genome-wide scale, however follow up studies characterizing the functional consequences of these binding events have lagged much further behind. By expanding the use of high-throughput reporter assays, we hope to advance our understanding of these functional consequences. This comprehensive comparison reveals the challenges and potential pitfalls of extrapolating binding events to transcriptional activation and shows the need for both approaches, as well as other experiments to truly characterize transcriptional activity.

## Methods

### Cloning, cell lines, and transfections of HaloTag vectors

Full-length human CREB1-α and -Δ cDNAs were obtained from OriGene, [NCBI:NM_134442.2 and NCBI:NM_004379.2], respectively. All CREB variants were subcloned into the pFN21A HaloTag CMV Flexi Vector (Promega) using SgfI and Pme, generating N-terminal HaloTag fusion constructs for each. HeLa cells (ATCC #CCL-2) were maintained in DMEM supplemented with 10%FBS at 37°C in an atmosphere of 5% CO_2_. Cells were transfected using Lipofectamine LTX transfection reagent (Invitrogen) according manufacturer's protocols.

### HaloCHIP Protocol and Whole Genome Amplification

A detailed version of the HaloCHIP protocol can be found at: 

For these experiments, HeLa cells (2-4 × 10^5^) were plated in a single well of a standard 6-well plate. After reaching 70-80% confluency, typically 18-24 hours later, cells were transfected with the HaloTag-CREB fusion constructs (experimental sample) or left untransfected (control sample). Twenty four hours post-transfection, cells were crosslinked with formaldehyde (Sigma) at a final concentration of 0.75% for 10 minutes at 22°, quenched with 0.125 M glycine for 10 min. and processed using the HaloCHIP kit (Promega). For experiments involving Forskolin, cells were treated with 10 μM Forskolin for 45 minutes at 37°C prior to crosslinking. Isolated DNA was further purified using a PCR Clean-up kit (Qiagen), and eluted 2 × 50 μl with nuclease-free water, yielding a final volume of 100 μl. To prepare sufficient HaloCHIP DNA for downstream amplification steps required for microarrays, an entire 6-well plate was transfected and processed through the HaloCHIP method as recommended. The isolated DNA was pooled before final purification on the PCR clean-up columns and lyophilized to a final volume of 12 μl. The concentrated HaloCHIP DNA was then amplified to 2-10 μg using the Whole Genome Amplification kit (Sigma) following the recommended adaptation for ChIP samples [46].

### ChIP Protocol

HeLa cells (4 × 10^6^) were plated in several 150 mm plates and grown at 37°C to 80-90% confluency. Cells were treated with 10 uM forskolin for 45 minutes at 37°. crosslinked with formaldehyde (Sigma) at a final concentration of 1.0% for 10 minutes at 22°, quenched with 0.125 M glycine for 10 min. and processed using the ChIP Assay Kit (USB). Chromatin was sheared by sonication using a Misonix MicroTip Probe 418, output of 5.5, with a program of 15 cycles of 5 seconds on and 25 seconds off on ice. Co-immunoprecipitation was performed using 1 μg of anti-CREB1 antibody (Millipore #06-863) for the experimental sample and 1 μg of anti-IgG antibody (Sigma) for the control sample with incubation at 4°C for 15 hours. Isolated DNA was further purified using a PCR Clean-up kit (Qiagen), processed, and amplified using WGA identical as the HaloCHIP samples.

### Quantitative PCR and primers

HaloCHIP DNA was analyzed using either Plexor (Promega) or SYBR green (Applied Biosystems) qPCR according to their respective manufacturer's recommendations. Plexor primers were supplied from Biosearch Technologies and SYBR green primers were from IDT DNA. The following sequences were used for amplification: **Fos **forward 5'-GTCTTGGCTTCTCAGATGCTCG-3', reverse 5'-GTTGAGCCCGTGACGTTTACA-3', **Jun **forward 5'-GAGAAAGAAGGGCCCGACTGT-3', reverse 5'-GGAGACTCCACCCTAGAAGATTCT-3', **p27 **forward 5'-GGGAGGCTGACGAAGAAGAAAAT-3', reverse 5'-CAACCAATGGATCTCCTCCTCTG-3', **C1 **forward 5'-CTGGTCTCACCTACCTTCCTGT-3', reverse 5'-ATCCATGAACTCCAGGAGCTCA-3' *C2 *forward 5'-TCTGTTGCCTATTGACCAGAACATG-3', reverse 5'-AGGAGCTGTAGGCTGAGTCAC-3', **C3 **forward 5'-CTGCTTCTTAACAGCTTAATTCGGAAGA-3', reverse 5'-ATGAGCAAAGATAGCTCAGGGAG-3'. Primers sequences used for PPV and NPV qPCR validation along with their corresponding amplified promoter can be found in supplemental materials: 

### Oligo array design and analysis

A custom oligo array was designed to cover a genome-wide set of human promoter regions predicted by SwitchGear Genomics (more detail can be found at ). The oligo array composed of approximately ~385,000 50mer probes was manufactured by Roche-NimbleGen Systems. The amplified enriched samples described above were shipped to Roche-NimbleGen to be hybridized according to their standard service protocol. The raw data from the arrays were analyzed as follows; the log2 ratio (enriched-cy5/total input-cy3) was calculated for each probe and data were then smoothed by averaging across a sliding window of 3 neighbouring probes shifting 1 probe at a time, minimizing noise from single probes. The median and standard deviation were calculated from the smoothed ratios for each sample. The median was subtracted from each ratio and divided by the standard deviation to center and normalize the data from each array. To summarize the enrichment for an entire promoter, the top 4 probe values were averaged for a given promoter region to approximate the 75^th ^percentile value. The raw data, normalized data, and collapsed data for each array are available as supplemental at the following site: . All microarray probes and data discussed in this publication have been deposited in NCBI's Gene Expression Omnibus[57] and are accessible through GEO Series accession number GSE18347 .

### Gene Ontology (GO) analysis

The Top 1% of the CREB HaloCHIP-chip promoters, 277 in total, were deposited and analyzed using Gene Ontology (GO)  and AmiGO as the search engine. GO categorized each promoter with respect to protein function, showed the number of promoters within each category, and reported a corresponding p-value based upon the calculations.

### High-throughput reporter assays and analysis

The promoter reporter assays used 235 promoter-reporter vectors (utilizing the *luc2P *reporter cassette from Promega) containing ~1 kb promoter fragments from known CREB-bound genes. These cloned promoters were selected from SwitchGear Genomic's genome-wide promoter clone collection (details on this panel of reporter constructs can be found at ). A panel of promoter controls was also used to normalize signals between plates and replicates. The 32 plate normalization controls, include ~1 kb fragments representing constitutively active human promoter fragments and random regions from the genome. The promoter reporter assay experiments were all conducted in 384-well format. A detailed protocol can be found at: . Transfection complexes were formed by incubating 50 ng of each individual promoter construct with 0.3 μL of Fugene 6 transfection reagent and Opti-MEM media in a total volume of 3 μL and incubated for 30 minutes. The co-transfection of the TORC1 expression construct was set up the same as the standard transfection reaction, but with the inclusion of 25 ng of TORC1 expression plasmid per reaction (TORC1 expression construct was provided by the Montminy lab). Transfection complexes were mixed with resuspended HeLa cells such that 4,000 HeLa cells were seeded in a volume of 50 μL in each well of a 384-well white tissue culture treated plate. Fifteen replicate wells of each promoter construct were performed representing triplicate assays in 5 different conditions: 1) no treatment, 2) PMA, 3) Forskolin, 4) TORC1, and 5) TORC1 + FSK.

After seeding and transfection, cells were incubated for 24 hours before inductions. Inductions were conducted for each plate by removing the old media and replacing with new media depending on the condition. For the untreated cells, fresh media was applied to each well. For the PMA induction, fresh media with 100 nM PMA was added to each well. For the forskolin induction, fresh media with 20 μM FSK was added to each well. Cells were kept in their respective induction condition for 4 hours and then frozen overnight at -80 degrees.

To read luminescent activity plates were thawed for 45 minutes at room temperature. Then 50 μL of Steady-Glo reagent (Promega #E2520) was added and incubated for 30 minutes at room temperature. Then luminescence was read for 2 seconds per well on a 384-well compatible plate luminometer (Molecular Devices LMax384).

The raw luminescent reads from each well were normalized as follows. Each 384 well plate contained 32 control wells that were comprised of 16 positive control promoters and 16 random genomic fragments that serve as background signal controls. These plate controls were used to normalize the per well values between plates within a condition. The average of the 3 replicates was taken, and the ratio of induced/untreated was calculated from the averages of the treated values and the untreated sample. A t-test for significance was also calculated between the 3 replicates of the induced and untreated samples. The background controls were also used to measure whether the average absolute signals were above background in each condition (>3 standard deviations from the mean of the negative controls). For a given promoter to be called induced or repressed it must pass the following criteria: 1) At least a 2-fold change (+/-) 2) Pass t-test with significance of p < 0.05 3) Must have absolute signals significantly above background.

## Competing interests

Promega Corporation sells the HaloCHIP system commercially, and SwitchGear Genomics sells promoter luciferase reporter vectors commercially. Some of the Promega authors hold stock in Promega Corporation, but less than 1% of such stock, and SwitchGear Genomics authors hold stock in SwitchGear Genomics. Promega Corporation and SwitchGear Genomics are the owners by assignment of patents or patent applications related to the HaloCHIP technology and the promoter reporter platform, respectively.

## Authors' contributions

DDH, JM, and NM carried out all HaloCHIP experiments and preparation of DNA for microarray analysis. NDT performed all array analysis and in conjunction with SFA carried out and analyzed all promoter macroarray analysis. DDH, NDT, KW, and MU conceived the study, planned experiments, and helped draft the manuscript. All authors read and approved the final manuscript.
